# The coming together of allosteric and phosphorylation mechanisms in the molecular integration of A2A heteroreceptor complexes in the dorsal and ventral striatal-pallidal GABA neurons

**DOI:** 10.1007/s43440-021-00314-3

**Published:** 2021-08-24

**Authors:** Dasiel O. Borroto-Escuela, Luca Ferraro, Sarah Beggiato, Manuel Narváez, Ramon Fores-Pons, Jose E. Alvarez-Contino, Karolina Wydra, Małgorzata Frankowska, Michael Bader, Małgorzata Filip, Kjell Fuxe

**Affiliations:** 1 Department of Neuroscience, Karolinska Institutet, Biomedicum (B0851), Solnavagen 9, 17177 Stockholm, Sweden; 2 Department of Life Sciences and Biotechnology, University of Ferrara, Ferrara, Italy; 3 Department of Medical, Oral and Biotechnological Sciences, University of Chieti-Pescara, 66100 Chieti, Italy; 4 Facultad de Medicina, Instituto de Investigación Biomédica de Málaga, Universidad de Málaga, Campus de Teatinos s/n, 29071 Málaga, Spain; 5 Policlínico Universitario Juan Bruno Zayas, Cifuentes, Villa Clara Cuba; 6 Department of Drug Addiction Pharmacology, Maj Institute of Pharmacology, Polish Academy of Sciences, 12 Smętna Street, 31343 Kraków, Poland; 7 Max-Delbrück-Centrum Für Molekulare Medizin (MDC), Robert-Rössle-Strasse 10, 13125 Berlin, Germany

**Keywords:** Allosteric receptor–receptor interactions, Adenosine A2A receptor, Cocaine, Striatal-enriched protein tyrosine phosphatase, Oligomerization, Phosphorylation

## Abstract

The role of adenosine A2A receptor (A2AR) and striatal-enriched protein tyrosine phosphatase (STEP) interactions in the striatal-pallidal GABA neurons was recently discussed in relation to A2AR overexpression and cocaine-induced increases of brain adenosine levels. As to phosphorylation, combined activation of A2AR and metabotropic glutamate receptor 5 (mGluR5) in the striatal-pallidal GABA neurons appears necessary for phosphorylation of the GluA1 unit of the AMPA receptor to take place. Robert Yasuda (J Neurochem 152: 270–272, 2020) focused on finding a general mechanism by which STEP activation is enhanced by increased A2AR transmission in striatal-pallidal GABA neurons expressing A2AR and dopamine D2 receptor. In his Editorial, he summarized in a clear way the significant effects of A2AR activation on STEP in the dorsal striatal-pallidal GABA neurons which involves a rise of intracellular levels of calcium causing STEP activation through its dephosphorylation. However, the presence of the A2AR in an A2AR-fibroblast growth factor receptor 1 (FGFR1) heteroreceptor complex can be required in the dorsal striatal-pallidal GABA neurons for the STEP activation. Furthermore, Won et al. (Proc Natl Acad Sci USA 116: 8028–8037, 2019) found in mass spectrometry experiments that the STEP splice variant STEP_61_ can bind to mGluR5 and inactivate it. In addition, A2AR overexpression can lead to increased formation of A2AR-mGluR5 heterocomplexes in ventral striatal-pallidal GABA neurons. It involves enhanced facilitatory allosteric interactions leading to increased Gq-mediated mGluR5 signaling activating STEP. The involvement of both A2AR and STEP in the actions of cocaine on synaptic downregulation was also demonstrated. The enhancement of mGluR5 protomer activity by the A2AR protomer in A2AR-mGluR5 heterocomplexes in the nucleus accumbens shell appears to have a novel significant role in STEP mechanisms by both enhancing the activation of STEP and being a target for STEP_61_.

## Introduction

In a recent Editorial by Robert Yasuda [1] on striatal-enriched protein tyrosine phosphatase (STEP) activity in central nervous system (CNS), the role of adenosine and STEP in modulating synaptic glutamate receptor function has been discussed, especially in relation to how STEP might be modulated by the adenosine A2A receptor (A2AR) activation [2]. Changes induced in striatal synaptic glutamate transmission were modulated by cocaine (10 µM) by an increase in adenosine levels and the consequent A2AR activation, a mechanism involving the participation of STEP [3] and mediating the A2AR agonist-induced reduction in cocaine self-administration. The possible involvement of A2AR-dopamine (DA) D2R heteroreceptor complex in STEP-mediated A2AR agonist-induced reduction in cocaine self-administration has also be proposed by R. Yasuda in its Editorial [1]. In this heteroreceptor complex, the A2AR protomer allosterically inhibits the DA D2R protomer signaling in the ventral striatal-pallidal GABA neurons [4, 5], leading to an inhibition of cocaine self-administration [6–8],

The present article aims to continue this original discussion, by focusing on the role that different A2AR heteroreceptor complexes might play in mediating STEP activation. In particular, the article will shortly illustrate the basic work on the neurochemical anatomy of the ventral and dorsal striatal-pallidal GABA neurons [4, 9, 10], and on adenosine receptor subtypes with a focus on A2AR, their signaling and function along with their heteroreceptor complexes [11, 12]. Then, the fundamentals of the STEP and its splice variants will be given [13–15], followed by presentation of STEP phosphorylation processes induced by activation of A2AR, metabotropic glutamate receptor 5 (mGluR5) and D2R.

Of special interest is the demonstration that upon activation by (S)-3,5-Dihydroxyphenylglycine (DHPG), a potent agonist of group I metabotropic glutamate receptors, STEP becomes activated in hippocampal slices and produces AMPA receptor (AMPAR) endocytosis [16]. The mGluR5 may therefore be involved in activating STEP. We now also propose that the A2AR protomer activation by the A2AR agonist in an A2AR-fibroblast growth factor receptor 1 (FGFR1) heteroreceptor complex [17] can be necessary for the STEP activation by A2AR stimulation.

## Striatal-pallidal GABA medium spiny neurons [MSNs; also known as spiny projection neurons (SPNs)]

### The dorsal striatal-pallidal GABA SPNs

These striatal GABA neurons are located in the caudate putamen, which forms the dorsal striatum representing a major part of the basal ganglia. These GABA neurons project into the external segment of globus pallidus, thus inhibiting the activity of pallidal-subthalamic nucleus (STN) GABA neurons, which in turn modulate the activity of STN glutamate pathway. STN glutamate projections then reach and drive the GABA projection neurons of the internal segment of globus pallidus and *pars reticulata* of the *substantia nigra*, which modulate the activity of the thalamic glutamate projections to the cortical motor regions, thus controlling movements [9, 18]. The striatal GABA neurons of this brain circuit are known as the indirect striatal projection neurons (i.e., striatal indirect pathway), since they connect indirectly to the internal segment of the globus pallidus and the *substantia nigra pars reticulata* via the external segment of the globus pallidus and the STN. About half of SPNs constitutes instead the direct pathway, projecting directly to the internal segment of the globus pallidus and *substantia nigra pars reticulata.*

Upon activation of the dorsal striatal-pallidal GABA neurons by glutamate synapses via the cortical-striatal glutamate neurons, inhibition of movements will develop through the brain circuit described above. In fact, an increased glutamate drive from the subthalamic nucleus will develop, leading to activation of the inhibitory GABA neurons of the internal segment of the globus pallidus and the *substantia nigra pars reticulata* projecting into the thalamus. This leads to an inhibition of the thalamic glutamate drive to the cortical motor regions. As result, movements will become reduced due to diminished action selection. Thus, activation of the dorsal striatal-pallidal GABA neurons represents the activation of a so-called “no-go” pathway [9].

The dorsal striatal-pallidal GABA pathway is enriched primarily in D2R and A2AR [9, 19]. Upon activation of the D2R located on the somato-dendritic regions of this pathway, the indirect SPN intrinsic excitability is inhibited through postsynaptic effects on ion channels, thus bringing down the impact of glutamate transmission and enhancing long-term synaptic depression development [18]. Thus, the D2R can upon agonist activation effectively reduce the activation of the indirect SPN and thereby improve movements, as also demonstrated by the evidence that D2R agonists possess antiparkinsonian actions [20].

### The ventral striatal-pallidal GABA SPNs

The main part of the ventral striatum is the nucleus accumbens, which is part of the basal ganglia and is innervated by the meso-limbic DA reward neurons [4, 6]. The nucleus accumbens connects with the ventral pallidum; the ventral striatal-ventral pallidum GABA SPNs originate from the nucleus accumbens shell and core. The ventral pallidum GABA projections then innerve the glutamate neurons of the medial dorsal thalamic nucleus which projects into the prefrontal cortex [4]. The analysis indicates that the ventral striatal-pallidal GABA SPNs are anti-reward and aversion neurons [6, 21, 22]. They are enriched in D2R and A2AR and the rewarding actions of cocaine can involve inhibition of the DA transporter with increased extracellular DA levels activating the D2R on the ventral striatal-pallidal GABA SPNs. These events lead to inhibition in activity of this anti-reward pathway which will enhance cocaine reward.

## Adenosine A2AR and their location and functional role in the dorsal and ventral striatum

Adenosine is an endogenous widespread modulator released in the CNS and periphery. Adenosine effects are mediated by four different subtypes of adenosine receptors: A1R, A2AR, A2BR and A3R [12]. All of them are high affinity receptors except the A2BR which are of low affinity. The A2ARs are Gs or Golf coupled receptors that operate via adenylate cyclase/protein kinase (AC/PKA) intracellular pathways involving activation of extracellular signal-regulated kinases (ERK1/2), protein kinase B (AKT) and cAMP response element-binding protein (CREB) through phosphorylation including also DA and cAMP-regulated phosphoprotein (DARPP-32)[23].

The A2ARs are highly expressed in the dorsal and ventral striatum and exist in reduced but significant densities *e.g.,* in the hippocampus and the cerebral cortical regions [12]. The A2AR is expressed both in neurons and in all types of astroglia. This adenosine receptor subtype has multiple functions in the CNS linked to movements, cocaine reward and addiction, excitotoxicity, learning and memory [12].

## Adenosine heteroreceptor complexes in the brain, especially in the dorsal and ventral striatum

The concept of direct receptor–receptor interactions in the plasma membrane between different types of receptors (especially G protein-coupled receptors; GPCRs) was introduced in the beginning of the 1980s and, in 1993, the concept was developed that they were made possible through the formation of receptor heterodimers or higher order heteromers [24, 25]. Subsequently, the term heteroreceptor complexes was introduced since it was not possible to know the composition and stoichiometry of the heteromers in tissues like the brain also involving the presence of various types of adapter proteins interacting with the GPCRs. Large numbers of homo- and heteroreceptor complexes have now been described in the brain [25]. They represent a new general integrative mechanism for multiple signals in the brain and other organs and, by allosteric receptor–receptor interactions, they modulate recognition, signaling and/or trafficking of the participating receptor protomers. Thus, in addition to the well-known phosphorylation mechanisms [26, 27], also allosteric mechanisms play a significant role in the heteroreceptor complexes. Receptor pharmacology and function become more diverse and selective through the allosteric receptor–receptor interactions in the heteroreceptor complexes.

### A2AR isoreceptor complexes

A2AR forms isoreceptor complexes when interacting with other types of adenosine receptor like A1R, A2BR and A3R [28–30], as demonstrated using proximity ligation assay [31–33] and BRET techniques [34]. A2AR can also interact with other A2ARs which leads to formation of A2AR-A2AR homodimers [34]. It is of substantial interest that *inter alia* the A2AR protomer can reduce the A1R protomer inhibitory signaling in the A1R-A2AR isoreceptor complex and the A2BR protomer can block the A2AR recognition and signaling in the A2AR-A2BR isoreceptor complex. It remains to be demonstrated which of them exist in the striatal-pallidal GABA neuron.

### A2AR heteroreceptor complexes

The most well-known A2AR heteroreceptor complex is the A2AR-D2R heteroreceptor complex demonstrated with *e.g.,* proximity ligation assay and BRET/FRET methods [33, 35], and found in highest densities in the dorsal and ventral striatal-pallidal GABA projection neurons [31, 32]. These heteroreceptor complexes likely also exist in the cholinergic striatal interneurons where the A2AR and D2R coexist. Antagonistic allosteric receptor–receptor interactions take place in these A2AR-D2R heterocomplexes through which agonist activation of the A2AR protomer produces a strong reduction of the affinity for the ligands on the high affinity D2R agonist binding of the D2R protomer. Furthermore, the A2AR agonist induces a strong reduction of the Gi/o-mediated signaling of the D2R signaling [35, 36]. The β-arrestin is instead recruited to the D2R protomer and takes over its signaling [37]. A structural model of the A2AR-D2R heterodimer was achieved with a transmembrane interface involving transmembrane IV/V domains [7, 34, 38]. These heteroreceptor complexes exist both in the ventral and dorsal striatal-pallidal GABA neurons, as detailed below.

#### Dorsal striatal-pallidal GABA neurons

It should be noted that an A2AR agonist blocks the D2R induced long-term depression with a return of long-term potentiation in the dorsal striatum. It likely involves antagonistic allosteric A2AR-D2R interaction in the dorsal striatal-pallidal GABA neurons [5]. The A2AR-D2R heteroreceptor complexes in these neurons are implicated in Parkinson’s disease and its treatment. Early on in this disease the motor dysfunction is effectively counteracted by levodopa treatment. However, with time, through the continued degeneration of especially the nigro-striatal DA neurons and continued chronic treatment with dopaminergic drugs, a reorganization of the A2AR-D2R heteroreceptor complexes likely develops. This can lead to reduction of the therapeutic effects of DA agonists involving enhanced pathology of the DA neurons and to augmented A2AR-mediated inhibition of the D2R signaling. Therefore, motor deficits increase with wearing off of the therapeutic effects of dopaminergic drugs.

#### Ventral striatal-pallidal GABA neurons

The nerve cell bodies of these projection neurons mediating anti-reward are mainly located in the nucleus accumbens and innervate the ventral pallidum. They are enriched in A2AR and D2R forming A2AR-D2R heteroreceptor complexes that bring down cocaine reward, as demonstrated in cocaine self-administration [6, 8, 21]. In cocaine use disorder (cocaine addiction), it was proposed that a permanent brake can develop on D2R protomer affinity and signaling in A2AR-D2R*-*Sigma1 receptor (Sigma1R) complexes formed upon chronic use of cocaine [6, 8, 39]. These pathological complexes can form a long-term memory involving a marked and long-lasting brake on D2R protomer function in the anti-reward GABA neurons, thus causing cocaine addiction. These heteroreceptor complexes may become new targets for treatment of cocaine addiction, involving receptor interface, interfering peptides and hetero-bivalent compounds that can remove the brake on D2R protomer signaling in the A2AR-D2R-Sigma1R complexes. In this way, the D2R protomer can improve its function and to some degree reduce the activity in the ventral striatal-pallidal GABA anti-reward neurons which will to a certain degree assist in returning cocaine reward.

#### A2AR-mGluR5 and A2A-D2R-mGluR5 heteroreceptor complexes

These heteroreceptor complexes likely exist both in the dorsal and ventral striatal-pallidal GABA neurons [40–42]. The A2AR and mGluR5 protomers synergize the effects of each other and to inhibit the D2R protomer signaling in this trimeric complex, leading to inhibition of the activity in the motor circuits regulated by the dorsal striatal-pallidal GABA neurons [11]. In the ventral striatal-pallidal GABA anti-reward neurons, these two receptor protomers again appear to enhance the signaling of each other and their downregulation of the D2R protomer signaling located in the same trimeric complex [41] leading to further inhibition of the inhibitory D2R protomer signaling. The activity of the GABA anti-reward neurons should, therefore, become further increased and cocaine reward further reduced.

The A2AR-D2R-mGluR5 heteroreceptor complexes, discussed above, in the ventral striatal-pallidal GABA anti-reward neurons are also of high relevance for understanding schizophrenia in view of the DA hypothesis of this disease [4, 43, 44]. In schizophrenic individuals, the A2AR and mGluR5 protomers can synergize to inhibit the overactivity of the D2R protomer signaling in the nucleus accumbens, which represents the major region of the ventral striatum.

#### A2AR-FGFR heteroreceptor complexes

In 2008, it was found by Flajolet et al. [17] that the FGFR1 can form a heteroreceptor complex with the A2AR in the dorsal striatal-pallidal GABA neurons using a yeast two-hybrid analysis. It enhanced structural and functional synaptic plasticity. Coactivation of the two receptors produced synergistic enhancement of spine and neurite densities and cortical-striatal long-term potentiation developed in the glutamate synapses on the striatal-pallidal GABA neurons.

As to the molecular mechanism, the authors proposed that the coactivation of the FGFR1 and A2AR protomers led to a synergistic in the ERK1/2/mitogen-activated protein kinase (MAPK) intracellular pathway [17]. In the case of the A2AR, the ERK1/2/MAPK pathway became increased through the AC/PKA/exchange protein directly activated by cAMP (EPAC)/Raf activation of MAPK. Instead, the FGFR1 became linked to the ERK1/2/MAPK pathway via the growth factor receptor-bound protein 2 (Grb2)/Ras/Raf-cascade. The heteromerization was regarded as necessary to confine the two receptor protomers to the same micro-environment that would allow the combined phosphorylation of a signaling molecule downstream. It should be noted that the stimulation of the ERK1/2/MAPK pathway was regarded as being controlled only by protein kinase phosphorylation actions. No indications for allosteric events in the heteroreceptor complex were obtained.

However, it was considered that protein phosphatases can have a role through dephosphorylation of inhibitory sites which would allow an optimal conformation of ERK1/2. Early on, it was in fact found that a protein phosphatase cascade can make possible converging glutamate and DA signals to stimulate striatal ERK1/2 [27]. It should be noted that a structural model of the A2AR-D2R heterodimer was achieved with a transmembrane interface involving transmembrane IV/V domains [7, 34, 38].

This early work opened up the possibility that phosphorylation and allosteric mechanisms can operate in parallel to integrate multiple transmitter and modulator signals in heteroreceptor complexes.

## On the role of STEP, a striatal-enriched protein tyrosine phosphatase

STEP targets a widespread network of synaptic and extra-synaptic brain proteins and is specific to the brain [45]. There exist a number of splice variants, STEP_61_, STEP_46_, STEP_38,_ and STEP_20._ They differ in terms of distribution in the brain, membrane association and length of protein. It should be noticed that only one of them is enriched in the striatum, namely STEP_46_. The long form STEP_61_ is present all over the brain and is expressed in high densities in the hippocampus and the cortex cerebri [15]. The STEP_61_ interactome is extensive and involves by 8% ion channels, receptors and transporters, by 20% vesicle trafficking proteins, by 7% scaffolding proteins, by 3% ubiquitin enzyme proteins, by 16% kinases and phosphatases, by 4% cell adhesion proteins, by 8% ATP synthase and ATPase, by 4% GPCR signaling proteins and by 30% cytoskeleton or-associated and motor proteins [13]. They are all regulated by STEP through dephosphorylation. It becomes clear that there exist multiple targets through which STEP_61_ can regulate synaptic and volume transmission.

It is of interest that STEP_61_ exists in high densities in the synaptic membranes but are diminished in the postsynaptic density [13]. It was suggested that STEP_61_ mainly may produce effects on its biological substrates in the extra-synaptic regions. These findings are of relevance since it indicates that also extra-synaptic receptors and their complexes mediating volume transmission [46] are regulated by STEP_61_.

As previously described, STEP_61_ represents the full-length protein. It has five different components, the proline-rich domain, the transmembrane domain, the kinase-interacting motif (KIM), the kinase-specificity sequence domain (KIS) and the protein tyrosine phosphatase domain (PTP) [15]. The four splice variants listed originate from a single gene but the STEP_38_ and STEP_20_ lack the PTP domain. Their function is, therefore, not clear since they miss the catalytic activity. It may be that these short splice STEP variants have the ability to compete with the long-splice STEP variants for the same STEP substrate-binding sites. There also exists another short STEP, namely STEP_33_, that is formed by truncation of STEP_61_ caused by calpain induced cleavage of STEP_61_ [15]_._

The major activation of STEP takes place through activated calcineurin turning on protein phosphatase 1, producing a dephosphorylation of STEP which causes activation of STEP [1]. As to removal of STEP, it was demonstrated that ubiquitination of STEP is produced by synaptic activation causing its degradation. This process is enhanced by the binding of STEP to postsynaptic density protein 95 (PSD-95). Furthermore, the PSD-95 stabilizes N-methyl-D-aspartate receptor (NMDAR) which improves its synaptic strength [47].

Furthermore, STEP differentially control NMDARs and AMPA receptors (AMPAR). In view of the ability of STEP to control many types of synaptic and extra-synaptic proteins, there is agreement that dysregulation of STEP can be a significant factor in contributing to synaptic and extra-synaptic plasticity. STEP can be a relevant factor in neurodegenerative diseases like Parkinson’s disease and there is support for the view that inhibition of STEP can be used in the treatment of neurocognitive disease [15, 48].

## Phosphorylation events in striatal-pallidal GABA neurons mediated via A2AR, mGluR5 and D2R

Svenningsson and colleagues in 1998 [49] found that A2ARs upon activation stimulate cyclic AMP-dependent phosphorylation of DARPP-32 in striatal-pallidal GABA neurons, leading to a conversion of this inactive molecule into a highly potent protein phosphatase 1 (PP1) inhibitor [50]. The DARPP-32/PP1 interaction regulates the strength of the AMPA and NMDA glutamate receptor signaling with their dephosphorylation reducing their currents over the ion channels [51]. It is of interest that the A2AR-induced activation of the PKA-DARPP-32 signaling leads to increased phosphorylation of the GluA1 unit of the AMPARs in the striatal-pallidal GABA neurons which is enhanced by a mGlu5R agonist [52]. It may develop through the existence of A2AR-mGlu5R and A2AR-mGluR5-D2R heteroreceptor complexes in the striatal-pallidal GABA neurons leading to enhancement of mGluR5 protomer signaling via facilitatory A2AR-mGluR5 allosteric interactions in these complexes [40, 53].

It should be noted that upon interference with the site for PKA phosphorylation at DARPP-32, the effects of the group 1 mGluR5 agonist (R, S)-3,5-dihydroxyphenylglycin (DHPG) on GluA1 phosphorylation was blocked [52]. Thus, there appears to exist a demand of increased phosphatase inhibition over the PKA-DARPP-32 pathway for enhanced phosphorylation of the GluA1 subunit of the AMPAR to develop. The demonstration that adenosine deaminase, which catalyzes the irreversible deamination of adenosine, blocked the Ser 845 phosphorylation of GluA1 by DHPG also shows the need for combined activation of A2AR and mGluR5 for the phosphorylation of GluA1 to take place [52]. In addition, it was found that combined agonist activation of mGluR5 and A2AR protomers appeared to enhance their allosteric inhibition of the D2R protomer that further reduces the inhibitory signaling of the D2R protomer [41, 53].

In the dorsal striatum, these events will lead to enhancement of the inhibition of the motor drive since the dorsal striatal-pallidal GABA neurons mediates inhibition of movements [11, 18, 54]. In line with these results, it was found early on that D2R antagonists enhance the GluA1 AMPAR phosphorylation related to the removal of the D2R mediated inhibition of the PKA/DARPP-32 signaling pathway [13].

## Understanding the link between the A2AR modulation of STEP activity and the A2AR protomer-induced enhancement of mGluR5 protomer signaling in A2AR-mGluR5 andA2AR-D2R-mGluR5 hetero complexes of the striatal-pallidal GABA neurons

In its Editorial [1], Robert Yasuda focused on having a general mechanism by which STEP is modulated by A2AR upon its activation in neurons expressing A2AR and D2R, which can include also neuroblastoma cell lines, known to express these receptors [55]. The modulation of STEP by A2AR involved a calcineurin/PP1 pathway which was calcium dependent [3]. Robert Yasuda in his abstract [1] summarizes in a clear way the significant effects of A2AR activation which involves a rise of intracellular levels of calcium, a STEP activation through dephosphorylation and an inhibition of synaptic signaling in ionotropic glutamate receptors by tyrosine dephosphorylation of these receptors. It leads to their removal from the plasma membrane through endocytosis and degradation by lysosomes.

**Fig. 1:**
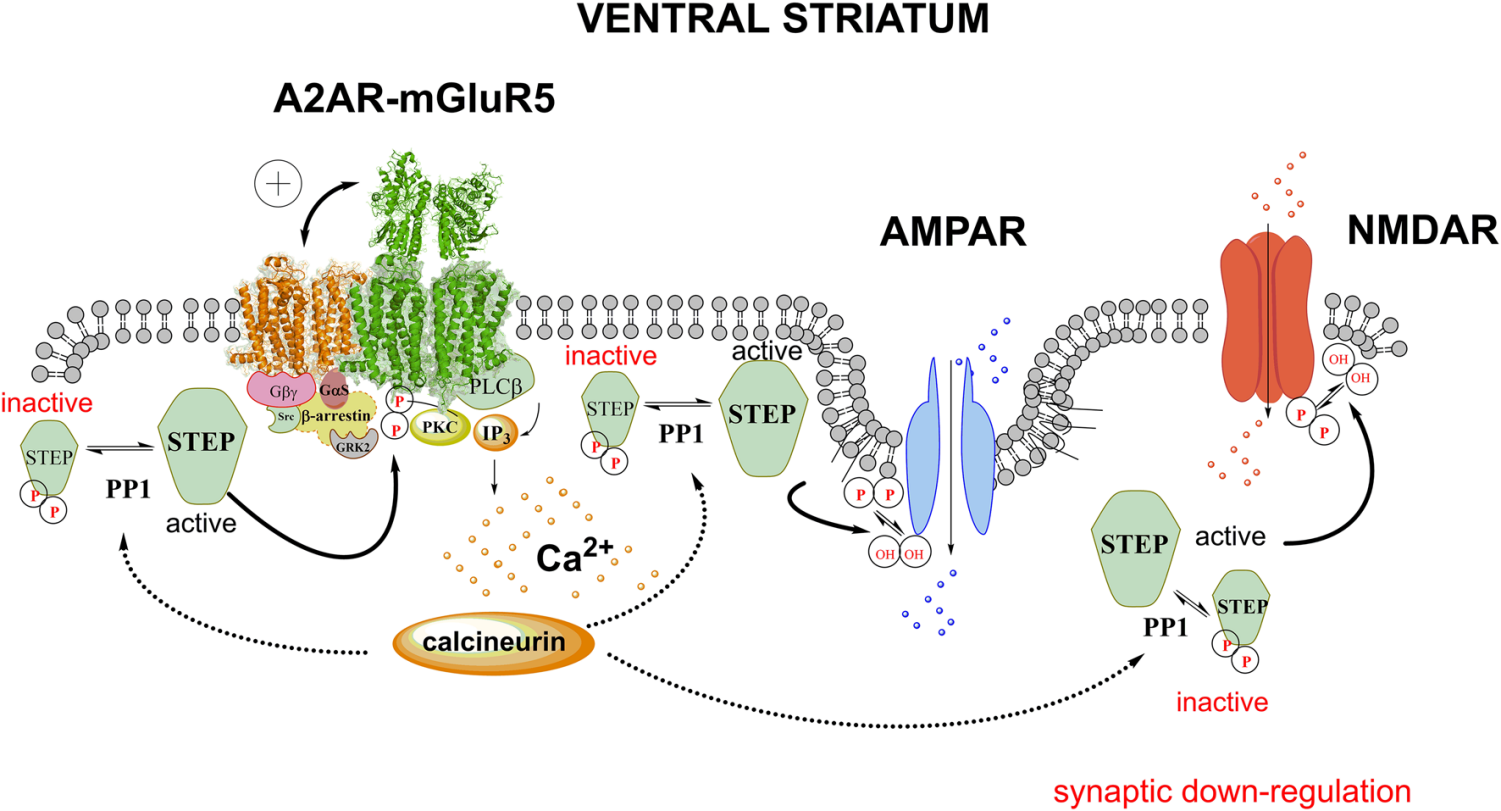
Cocaine, A2AR and STEP interactions activating STEP in the ventral striatum can be increased through enhanced allosteric receptor-receptor interactions in A2AR-mGluR5 heteroreceptor complexes in the ventral striatal-pallidal GABA neurons with A2AR protomer (orange color) activation increasing mGluR5 protomer (green color) signaling. The Gq activation results in increased PLC beta activity leading to a rise of intracellular calcium levels via improved IP3 levels and increased activation of calcineurin. Its phosphatase activity in turn activates PP1 which then via dephosphorylation activates STEP. Activated STEP bound to AMPAR and NMDAR can then inactivate these excitatory synaptic glutamate receptors via dephosphorylation followed by their internalization. A dynamic synaptic downregulation develops. To the left part of the figure, it is shown that this dynamic process can be counteracted by PP1induced activation of STEP bound to mGluR5 leading to its inactivation. The reduction of the mGluR5 signaling can then reduce the process of activated STEP binding to AMPAR and NMDAR with a return of excitatory synaptic signaling

Recently, in mass spectrometry experiments, it was found that the STEP splice variant STEP_61_ can bind to a large network of synaptic proteins [13]. Of high relevance for the current perspective article is that the STEP_61_ interactome involves not only GluN2B, GluN1 but also GluA2, GluA3 subunits of the NMDAR and AMPAR, respectively, and especially mGluR5 but not GluA1 [13]. Thus, the synergistic A2AR-mGluR5 receptor–receptor interactions [40] enhancing their signaling become of substantial interest not only because the Gq signaling of mGluR5 produces increases of intracellular calcium levels leading to activation of STEP (Domenici et al., 2021), but also because there is a direct STEP feedback onto the mGluR5 (Fig. 1). This can produce a dephosphorylation of its tyrosine residues with hypofunction of mGluR5 regarding *e.g.*, interplay between GluN and mGluR5, evoked ERK1/2 signaling and the allosteric A2AR-mGluR5 interactions [56–58]. It will also be of substantial interest to study how the binding of STEP_61_ and other splice variants of STEP through their dephosphorylation actions can modulate the striatal A2AR-mGluR5 and A2AR-D2R-mGluR5 heteroreceptor complexes. It should be noticed that the work of Won et al. [13] was performed on hippocampus and neocortex where STEP_61_ is by far the dominating form.

The most frequent AMPAR is built up of a GluA1/2 heteromer [13]. In view of the absence of STEP_61_ binding to the GluA1 [13], the direct STEP_61_ modulation of this AMPAR may only involve tyrosine dephosphorylation of the GluA2 unit but with a potential for allosteric modulation by the dephosphorylated GluA2 of the GluA1 subunit. However, it should be considered that other splice variants of STEP may bind to GluA1.

It is of special interest that in transgenic rats overexpressing neuronal A2AR in the brain under the regulation of a neural-specific enolase promotor [59, 60], markedly enhanced increases developed in protein tyrosine phosphatases and especially in STEP compared to control rats taking place *inter alia* in the striatum [2]. This increase appeared to be maximal since an A2AR agonist failed to increase it further, but the increase was blocked by an A2AR antagonist. The signaling pathway may involve an A2AR-mediated increase in intracellular calcium levels that likely involves the A2AR-mGluR5 heteroreceptor complex with A2AR-induced enhancement of Gq signaling of the mGluR5 increasing phospholipase C (PLC) activity and intracellular calcium levels. This leads to activation of calcineurin which via PP1 can activate STEP through its dephosphorylation [2] (Fig. 1). This produces synaptic depression through dephosphorylation of critical tyrosine phosphates, located on the C terminal parts of NMDARs and AMPARs which leads to a differential regulation of NMDAR and AMPAR internalization [1, 13]. Inhibition of the function of the ERK1/2 signaling pathway also takes place [1]. It was concluded that STEP_61_ has the power of being a key regulator of synaptic glutamate receptors through dephosphorylation, enhancing internalization of AMPAR and NMDAR in extra-synaptic regions [13].

It should be considered that these events can take place in the same neuron of the A2AR, mGluR5 and D2R enriched striatal-pallidal GABA pathway that also possess the A2AR-D2R heteroreceptor complexes in balance with A2AR-mGluR5 and A2AR-D2R-mGluR5 heteroreceptor complexes and the corresponding homoreceptor complexes [8, 11, 40, 41]. A2AR activation puts a brake on D2R protomer recognition and signaling but enhances mGluR5 signaling and their facilitatory allosteric interactions leading to enhanced inhibition of D2R function [55] that is known to inhibit this pathway [18, 54]. This marked inhibition of D2R signaling produces a substantial loss of inhibition of the striatal-pallidal GABA neurons leading to overactivity that in its dorsal component produces inhibition of movements due to the increased firing of this pathway [18, 54]. In contrast, a strong coupling of A2AR/mGluR5 signaling of these neurons to STEP activation results in synaptic depression of this pathway. It is due to marked loss of synaptic AMPA and NMDA glutamate receptor function through dephosphorylation [1] with return of motor activity since the synaptic glutamate receptor drive is no longer in operation at this time in this pathway [1].

The increased strength of STEP activation upon overexpression of neuronal A2AR in brain regions, including the striatum, of a transgenic rat strain can be explained by enhanced production of other types of A2AR complexes like A2A homoreceptor complexes built up of two or more A2AR protomers and A2AR-D2R heteroreceptor complexes with a stoichiometry dominated by A2AR protomers. As a result, high activity A2AR homomers with a lack of inhibitory allosteric inputs from the D2R protomer can be formed. However, we favor the impact of enhanced formation of A2AR-mGluR5 heteroreceptor complexes that can develop with increases in signaling and coupling of the mGluR5 protomer to Gq linked to producing activation of PLC leading to STEP activation [1, 2]. Novel types of adapter proteins within the A2AR-mGluR5 heteroreceptor complexes may also participate and enhance the mGluR5 protomer signaling.

Such events may help explain the markedly increased ability of overexpressed A2AR to strongly activate STEP, namely through enhanced mGluR5 signaling activating STEP leading to marked dephosphorylation by STEP of synaptic glutamate receptors followed by endocytosis in extra-synaptic domains. Thus, signs of synaptic glutamate depression can develop due to a reduction of synaptic expression of excitatory NMDAR and AMPAR with their currents mediating the excitatory synaptic glutamate transmission in which the dephosphorylation of mGluR5 by STEP can also play a relevant role [1, 2, 13].

## Cocaine modulation of A2A-homo- and heteroreceptor complexes and their link to STEP in the ventral and dorsal striatal-pallidal GABA neurons

Cocaine was early on shown to interact directly with the Sigma1R [61, 62] and the Sigma1R agonist OSU-6162 enhanced the antagonistic A2A-D2R receptor–receptor interactions in cocaine self-administration [8, 39]. A2AR agonists and Sigma1R agonists appear to synergize in their inhibition of D2R protomer signaling leading to increased firing in the ventral striatal-pallidal anti-reward pathways, strongly contributing to inhibition of cocaine reward and cocaine seeking [6, 21].

As pointed out by Robert Yasuda [1], it is of substantial interest that disruption of the A2AR-D2R heteroreceptor complex in the nucleus accumbens fully counteracts the A2AR agonist inhibition of cocaine self-administration [7]. Thus, this heteroreceptor complex is required for the ability of A2AR agonist to inhibit cocaine self-administration, which is mediated through the inhibition of the D2R protomer recognition and signaling [6, 21]

The differential role of the A2AR in the dorsal *versus* ventral striatum will be discussed with regard to STEP activity. The significant work of Puighermanal et al. [10] should again be considered. They found highly interesting functional and molecular heterogeneities among D2R-positive neuronal populations along the dorsal ventral axis in the striatum, which could be linked to specific motor behaviors like digging behavior and amphetamine-mediated hyperlocomotion [10]. The existence of D2R populations within the dorsal and ventral striatal-pallidal GABA neurons with a distinct pattern of dynamic heteroreceptor complexes involving *inter alia* different types of A2AR heteroreceptor complexes, could contribute to these molecular heterogeneities and help define subgroups of D2R-positive striatal neurons with distinct functions.

### Dorsal striatum

In agreement with Robert Yasuda opinion [1], it is of high interest that cocaine can reduce the excitatory postsynaptic currents mediated by AMPAR and NMDAR in dorsal striatum [3]. The ion currents were counteracted by an A2AR antagonist and a trapping mutant of the STEP substrate. Therefore, the A2A receptor activation appears to modulate the activity of the STEP in neuronal cells [14]. Thus, the results indicated the involvement of both A2AR and STEP in the actions of cocaine leading to synaptic glutamate receptor downregulation.

Other results in the dorsal striatum on the effects of cocaine self-administration on the A2AR-D2R and A2AR-mGluR5 heteroreceptor complexes [8] would be compatible with the results above in the dorsal striatum [3]. Thus, there was a trend for a reduction of the density of the A2AR-D2R and a non-significant reduction in the density of the A2AR-mGluR5 heteroreceptor complexes in the dorsal striatum following cocaine self-administration. Furthermore, cocaine self-administration induced a significant reduction in the density of D2R-Sigma1R heteroreceptor complexes in the dorsal striatum [8]. The trends for reductions in the density of both the A2AR-D2R and A2AR-mGluR5 hetero complexes opened the possibility that an increased number of A2ARs became free to bind to other A2AR, thus increasing the density of A2AR-A2AR homoreceptor complexes. Such types of increases in A2AR homoreceptor complexes and A2AR monomers in the dorsal striatum may then contribute to the demonstrated A2AR-induced activation of STEP [3].

As to the mechanism for the activation by the A2AR of STEP in the dorsal striatum, it is suggested that its actions can involve and become enhanced by its participation in A2AR-FGFR1 heteroreceptor complexes in this region [17] (Fig. 1). Thus, coactivation of these two receptor protomers, but not their single activation, resulted in a strong increase in the activity of the MAPK-ERK1/2 signaling pathway [17]. This can in part be mediated by positive allosteric receptor-receptor interactions in these A2AR-FGFR1 heteroreceptor complexes. Co-stimulation also led to cortical-striatal long-term potentiation in the dorsal striatal-pallidal GABA neurons and to marked structural changes in *e.g.*, neurite outgrowth. A marked phosphorylation of ERK1/2 developed which was rapid and long-lasting.

Furthermore, cocaine produced ERK1/2 activation in the striatum, an action which was counteracted by point mutation at Thr-34 in DARPP-32. This protein is known to mediate inhibition of PP1 and to be activated by A2AR-PKA signaling in the striatal-pallidal GABA neurons [27]. DARPP-32 regulation can take place both at the ERK1/2 and STEP level. However, DARPP-32 appeared to control STEP activation in a negative way [27]. Upon phosphorylation by PKA, DARPP-32 inhibited the PP1 activity and phosphorylated STEP (p-STEP) was not dephosphorylated and thus not activated. However, the activated A2AR protomer can have enhanced, via an allosteric mechanism, the FGFR1 protomer signaling in the A2AR-FGFR1 heteroreceptor complex in the dorsal striatum. Activated FGFR1 can then accumulate in the nucleus and interact with the transcriptional co-activator CREB-binding protein leading to gene activation (Fig. 1) [63]. Consequently, a new panorama of proteins can be formed that can have protein phosphatase activity and replace the role of PP1 in activating p-STEP. Alternatively, the protein can have stimulatory effects on PP1 instead of inhibitory actions as exerted by P-Thr -DARPP-32. Future work in the dorsal striatum should test this mechanism as a way for A2AR to increase STEP activity.

### Ventral striatum

As already discussed, cocaine was early on shown to act directly with the Sigma1R [61, 62]. It is important to note that cocaine administration selectively increased Sigma1R in the ventral striatum, but not in the dorsal striatum [64]. A2AR agonists and Sigma1R agonists appear to synergize in their inhibition of D2R protomer signaling leading to increased firing in the ventral striatal-pallidal anti-reward pathways, strongly contributing to inhibition of cocaine reward and cocaine seeking [6, 21]. Thus, A2AR-D2R-Sigma1R heteroreceptor complexes likely are formed in the nucleus accumbens shell, but not in the dorsal striatum, upon cocaine self-administration [8].

In fact, in the work of Borroto-Escuela et al. [8] studying the effects of cocaine self-administration on the A2AR-D2R and A2AR-mGluR5 heteroreceptor complexes in the nucleus accumbens versus the dorsal striatum, marked differences were observed in their responses to cocaine self-administration. In marked contrast to dorsal striatum, a significant increase of the A2AR-D2R complexes and a trend for an increase of the A2AR-mGluR5 complexes were observed in the nucleus accumbens shell. These results indicated that in this part of the nucleus accumbens the balance of the density of A2AR heteroreceptor complexes with the density of the A2AR-A2AR homoreceptor complexes had switched towards a dominance versus the A2AR heteroreceptor complexes. Thus, upon cocaine self-administration a reduced density of A2AR homomers and A2AR monomers should exist. A possible STEP activation can in this case not be explained by an increase in the density of A2AR homomers-monomers.

Should a STEP activation develop in the nucleus accumbens shell upon cocaine self-administration, it may instead be related to the trend for an increase in the A2AR-mGluR heteroreceptor complexes [8]. In addition, it is known that these two receptor protomers allosterically enhance the effects of each other upon coactivation via allosteric receptor-receptor interactions [40]. Thus, allosteric enhancement of the mGluR5 signaling through activation of the A2AR protomers can take place. In this way, the Gq signaling of the mGluR5 protomer will become enhanced leading to increased intracellular levels of calcium that via calcineurin/PP1 activation can activate STEP.

This proposal is in line with the significant work of Won et al. [13] demonstrating that following STEP_61_ overexpression and activation, the phosphatase can directly bind to NMDARs and indirectly to AMPARs via transmembrane regulatory proteins enhancing their internalization at extra-synaptic sites which may be true also for mGluR5 [13]. Thus, at least this splice variant of STEP can bind to mGluR5 and dephosphorylate its tyrosine residues leading to reduction of its activation with negative consequences for the phosphorylation of AMPAR and NMDAR [52].

These results suggested the existence of a highly dynamic balance between the activity of A2AR-mGluR5 heteroreceptor complexes and the activity of STEP in the ventral but not dorsal striatal-pallidal GABA neurons which increase and reduce glutamate synaptic function, respectively. According to the results above, the mGluR5 protomer in A2AR-mGluR5 heteroreceptor complexes seems to have an especially significant role by both enhancing the activation of STEP and being a target for STEP producing a downregulation of mGluR5 in the nucleus accumbens shell. mGluR5 should contribute to a dynamic up- and downregulation of synaptic NMDAR (GluN2B and GluN1) and AMPAR (GluA2 and GluA3), which may be crucial for the dynamic regulation of the brain networks and their function, including learning and memory.

**Fig. 2:**
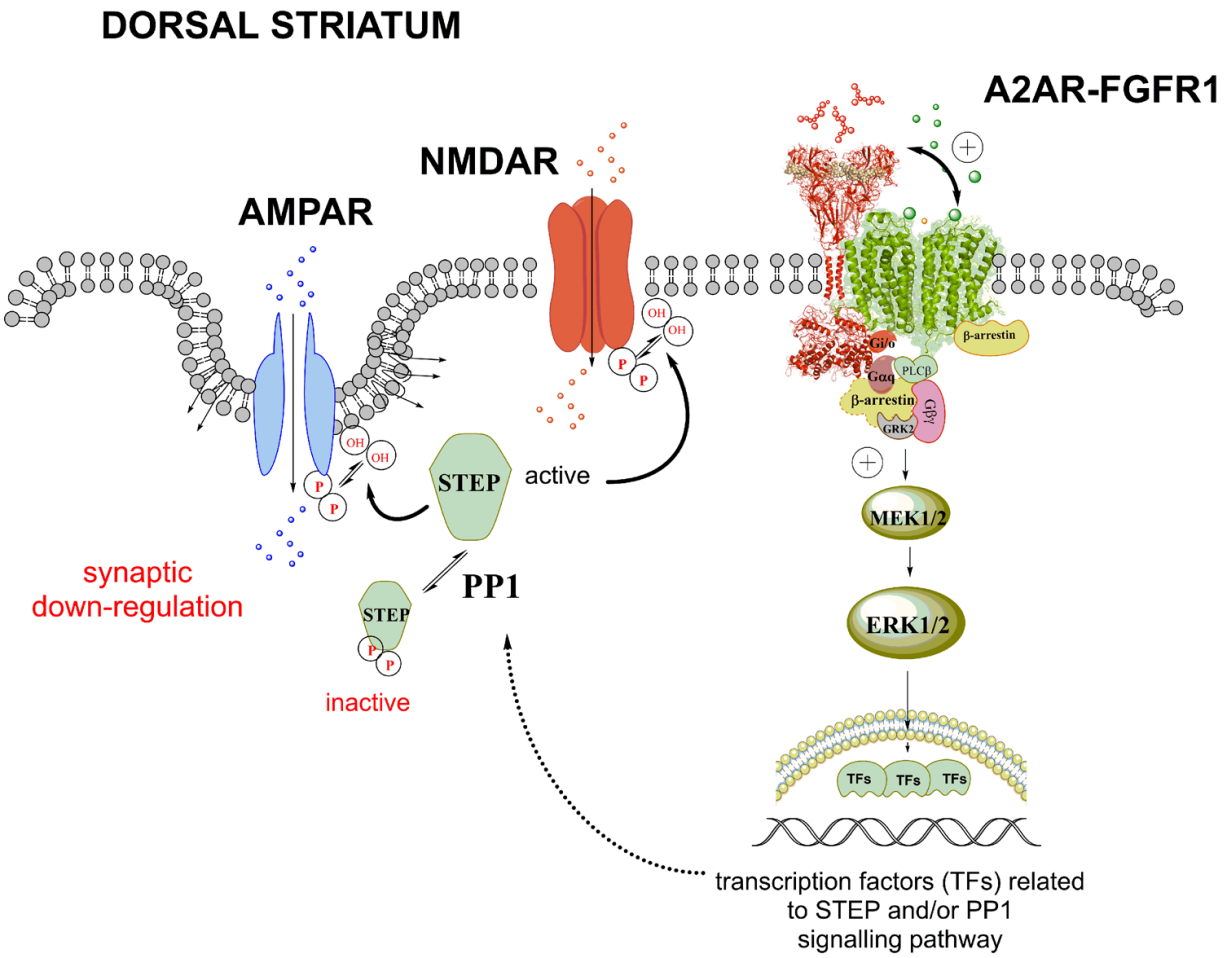
Cocaine, A2AR and STEP interactions activating STEP in the dorsal striatum can be increased through enhanced allosteric receptor–receptor interactions in A2AR-FGFR1 heteroreceptor complexes in the dorsal striatal-pallidal GABA neurons with the A2AR protomer activation increasing FGFR1 signaling. In particular, the enhanced activity of the RAS/MAPK pathway (ERK1/2) of FGFR1 can activate transcription factors linked to the formation of STEP, PP1 and/or their interacting proteins. As a result of these transcription factors, enhanced activation of STEP can develop which can be one mechanism for the ability of the A2AR protomer to enhance STEP activity in the dorsal striatal-pallidal GABA pathway. Thus, an increased excitatory synaptic down-regulation can develop in these GABA neurons

However, it is a complex regulation that can differ from one brain region to another one like dorsal striatum versus ventral striatum. The results indicated [8] that in the nucleus accumbens shell the A2AR-mGluR5 heteroreceptor complexes can have a significant role in cocaine self-administration involving activation of STEP function through A2AR protomer-induced enhancement which can involve of mGluR5 protomer signaling. This mechanism is different from the one in dorsal striatum where Chiodi et al. [3] demonstrated for the first time that A2AR activation can increase STEP function. As discussed, in this region the mechanism can be the activation of the A2AR protomer in the A2AR-FGFR1 heteroreceptor complex in the dorsal striatum (Fig. 2).

## Conclusions

Recent works have demonstrated the existence of significant interactions between cocaine, A2AR, and STEP involving A2AR overexpression and cocaine actions in the dorsal striatum [1–3]. The mechanism for STEP activation can involve the activation of the A2AR protomer in an A2AR-FGFR1 heteroreceptor complex in the dorsal striatum with allosteric enhancement also of the FGFR1 protomer signaling. The work of Won et al. [13] has also shown that mGluR5 can be a target for STEP. It has previously not been discussed how mGluR5 can contribute to understanding cocaine, A2AR and STEP interactions in the ventral striatal-pallidal GABA anti-reward neurons. It appears as if the mGluR5 protomer in A2AR-mGluR5 hetero complexes and its Gq-mediated signaling in the nucleus accumbens shell can play an important role in activating STEP. It can participate in mediating the STEP activation by cocaine in parts of the ventral striatum. It is proposed that A2AR-mGluR5 heteroreceptor complexes can play a significant role through A2AR-mediated allosteric enhancement of Gq-mediated mGluR5 signaling in this complex. In view of the fact that STEP_61_ can directly bind to mGluR5 [13], it will be of high interest to test how mGluR5 can play a key role in the dynamic balance with STEP activation. mGluR5 appears to significantly modulate through phosphorylation and allosteric mechanisms the synaptic activity of AMPA and NMDA receptors. These actions by mGluR5 can involve the modulation of the dephosphorylation of these synaptic glutamate receptors by activated STEP associated with AMPA and NMDA receptors which can lead to synaptic depression in the nucleus accumbens shell.

## References

[1] Yasuda RP (2020). Adenosine STEPs on synaptic function: an editorial for 'the activity of the STriatal-enriched protein tyrosine phosphatase in neuronal cells is modulated by adenosine A2A receptor on' page 284. J Neurochem.

[2] Mallozzi C, Pepponi R, Visentin S, Chiodi V, Lombroso PJ, Bader M, Popoli P, Domenici MR (2020). The activity of the Striatal-enriched protein tyrosine phosphatase in neuronal cells is modulated by adenosine A2A receptor. J Neurochem.

[3] Chiodi V, Mallozzi C, Ferrante A, Chen JF, Lombroso PJ, Di Stasi AM, Popoli P, Domenici MR (2014). Cocaine-induced changes of synaptic transmission in the striatum are modulated by adenosine A2A receptors and involve the tyrosine phosphatase STEP. Neuropsychopharmacology.

[4] Fuxe K, Marcellino D, Rivera A, Diaz-Cabiale Z, Filip M, Gago B, Roberts DC, Langel U, Genedani S, Ferraro L, de la Calle A, Narvaez J, Tanganelli S, Woods A, Agnati LF (2008). Receptor-receptor interactions within receptor mosaics. Impact Neuropsychopharmacol Brain Res Rev.

[5] Borroto-Escuela DO, Hinz S, Navarro G, Franco R, Muller CE, Fuxe K (2018). Understanding the role of adenosine A2AR heteroreceptor complexes in neurodegeneration and neuroinflammation. Front Neurosci.

[6] Borroto-Escuela DO, Wydra K, Filip M, Fuxe K (2018). A2AR-D2R heteroreceptor complexes in cocaine reward and addiction. Trends Pharmacol Sci.

[7] Borroto-Escuela DO, Wydra K, Li X, Rodriguez D, Carlsson J, Jastrzebska J, Filip M, Fuxe K (2018). Disruption of A2AR-D2R heteroreceptor complexes after A2AR transmembrane 5 peptide administration enhances cocaine self-administration in rats. Mol Neurobiol.

[8] Borroto-Escuela DO, Narvaez M, Wydra K, Pintsuk J, Pinton L, Jimenez-Beristain A, Di Palma M, Jastrzebska J, Filip M, Fuxe K (2017). Cocaine self-administration specifically increases A2AR-D2R and D2R-sigma1R heteroreceptor complexes in the rat nucleus accumbens shell. Relevance for cocaine use disorder. Pharmacol Biochem Behav.

[9] Surmeier DJ, Graves SM, Shen W (2014). Dopaminergic modulation of striatal networks in health and Parkinson's disease. Curr Opin Neurobiol.

[10] Puighermanal E, Castell L, Esteve-Codina A, Melser S, Kaganovsky K, Zussy C, Boubaker-Vitre J, Gut M, Rialle S, Kellendonk C, Sanz E, Quintana A, Marsicano G, Martin M, Rubinstein M, Girault JA, Ding JB, Valjent E (2020). Functional and molecular heterogeneity of D2R neurons along dorsal ventral axis in the striatum. Nat Commun.

[11] Borroto-Escuela DO, Fuxe K (2019). Adenosine heteroreceptor complexes in the basal ganglia are implicated in Parkinson's disease and its treatment. J Neural Transm.

[12] Borea PA, Gessi S, Merighi S, Vincenzi F, Varani K (2018). Pharmacology of adenosine receptors: the state of the art. Physiol Rev.

[13] Won S, Incontro S, Li Y, Nicoll RA, Roche KW (2019). The STEP61 interactome reveals subunit-specific AMPA receptor binding and synaptic regulation. Proc Natl Acad Sci USA.

[14] Domenici MR, Mallozzi C, Pepponi R, Casella I, Chiodi V, Ferrante A, Popoli P (2021). Insight into the role of the striatal-enriched protein tyrosine phosphatase (STEP) in A2A receptor-mediated effects in the central nervous system. Front Pharmacol.

[15] Won S, Roche KW (2021). Regulation of glutamate receptors by striatal-enriched tyrosine phosphatase 61 (STEP61). J Physiol.

[16] Zhang Y, Venkitaramani DV, Gladding CM, Zhang Y, Kurup P, Molnar E, Collingridge GL, Lombroso PJ (2008). The tyrosine phosphatase STEP mediates AMPA receptor endocytosis after metabotropic glutamate receptor stimulation. J Neurosci Off J Soc Neurosci.

[17] Flajolet M, Wang Z, Futter M, Shen W, Nuangchamnong N, Bendor J, Wallach I, Nairn AC, Surmeier DJ, Greengard P (2008). FGF acts as a co-transmitter through adenosine A(2A) receptor to regulate synaptic plasticity. Nat Neurosci.

[18] Zhai S, Shen W, Graves SM, Surmeier DJ (2019). Dopaminergic modulation of striatal function and Parkinson's disease. J Neural Transm.

[19] Stromberg I, Popoli P, Muller CE, Ferre S, Fuxe K (2000). Electrophysiological and behavioural evidence for an antagonistic modulatory role of adenosine A2A receptors in dopamine D2 receptor regulation in the rat dopamine-denervated striatum. Eur J Neurosci.

[20] Fuxe K, Hall H, Kohler C (1979). Evidence for an exclusive localization of 3H-ADTN binding sites to postsynaptic nerve cells in the striatum of the rat. Eur J Pharmacol.

[21] Wydra K, Gawlinski D, Gawlinska K, Frankowska M, Borroto-Escuela DO, Fuxe K, Filip M (2020). Adenosine A2A receptors in substance use disorders: a focus on cocaine. Cells.

[22] Koob GF, Le Moal M (2001). Drug addiction, dysregulation of reward, and allostasis. Neuropsychopharmacol.

[23] Preti D, Baraldi PG, Moorman AR, Borea PA, Varani K (2015). History and perspectives of A2A adenosine receptor antagonists as potential therapeutic agents. Med Res Rev.

[24] Zoli M, Agnati LF, Hedlund PB, Li XM, Ferre S, Fuxe K (1993). Receptor-receptor interactions as an integrative mechanism in nerve cells. Mol Neurobiol.

[25] Borroto-Escuela DO, Brito I, Romero-Fernandez W, Di Palma M, Oflijan J, Skieterska K, Duchou J, Van Craenenbroeck K, Suarez-Boomgaard D, Rivera A, Guidolin D, Agnati LF, Fuxe K (2014). The G protein-coupled receptor heterodimer network (GPCR-HetNet) and its hub components. Int J Mol Sci.

[26] Greengard P, Nairn AC, Girault JA, Ouimet CC, Snyder GL, Fisone G, Allen PB, Fienberg A, Nishi A (1998). The DARPP-32/protein phosphatase-1 cascade: a model for signal integration. Brain Res Brain Res Rev.

[27] Valjent E, Pascoli V, Svenningsson P, Paul S, Enslen H, Corvol JC, Stipanovich A, Caboche J, Lombroso PJ, Nairn AC, Greengard P, Herve D, Girault JA (2005). Regulation of a protein phosphatase cascade allows convergent dopamine and glutamate signals to activate ERK in the striatum. Proc Natl Acad Sci USA.

[28] Ciruela F, Casado V, Rodrigues RJ, Lujan R, Burgueno J, Canals M, Borycz J, Rebola N, Goldberg SR, Mallol J, Cortes A, Canela EI, Lopez-Gimenez JF, Milligan G, Lluis C, Cunha RA, Ferre S, Franco R (2006). Presynaptic control of striatal glutamatergic neurotransmission by adenosine A1–A2A receptor heteromers. J Neurosci.

[29] Hinz S, Navarro G, Borroto-Escuela D, Seibt BF, Ammon YC, de Filippo E, Danish A, Lacher SK, Cervinkova B, Rafehi M, Fuxe K, Schiedel AC, Franco R, Muller CE (2018). Adenosine A2A receptor ligand recognition and signaling is blocked by A2B receptors. Oncotarget.

[30] Borroto-Escuela DO, Carlsson J, Ambrogini P, Narvaez M, Wydra K, Tarakanov AO, Li X, Millon C, Ferraro L, Cuppini R, Tanganelli S, Liu F, Filip M, Diaz-Cabiale Z, Fuxe K (2017). Understanding the role of GPCR heteroreceptor complexes in modulating the brain networks in health and disease. Front Cell Neurosci.

[31] Zhu Y, Dwork AJ, Trifilieff P, Javitch JA (2020). Detection of G protein-coupled receptor complexes in postmortem human brain by proximity ligation assay. Curr Protoc Neurosci.

[32] Trifilieff P, Rives ML, Urizar E, Piskorowski RA, Vishwasrao HD, Castrillon J, Schmauss C, Slattman M, Gullberg M, Javitch JA (2011). Detection of antigen interactions ex vivo by proximity ligation assay: endogenous dopamine D2-adenosine A2A receptor complexes in the striatum. Biotechniques.

[33] Borroto-Escuela DO, Romero-Fernandez W, Garriga P, Ciruela F, Narvaez M, Tarakanov AO, Palkovits M, Agnati LF, Fuxe K (2013). G protein-coupled receptor heterodimerization in the brain. Methods Enzymol.

[34] Borroto-Escuela DO, Rodriguez D, Romero-Fernandez W, Kapla J, Jaiteh M, Ranganathan A, Lazarova T, Fuxe K, Carlsson J (2018). Mapping the interface of a GPCR dimer: a structural model of the A2A adenosine and D2 dopamine receptor heteromer. Front Pharmacol.

[35] Borroto-Escuela DO, Marcellino D, Narvaez M, Flajolet M, Heintz N, Agnati L, Ciruela F, Fuxe K (2010). A serine point mutation in the adenosine A2AR C-terminal tail reduces receptor heteromerization and allosteric modulation of the dopamine D2R. Biochem Biophys Res Commun.

[36] Azdad K, Gall D, Woods AS, Ledent C, Ferre S, Schiffmann SN (2009). Dopamine D2 and adenosine A2A receptors regulate NMDA-mediated excitation in accumbens neurons through A2A–D2 receptor heteromerization. Neuropsychopharmacology.

[37] Borroto-Escuela DO, Romero-Fernandez W, Tarakanov AO, Ciruela F, Agnati LF, Fuxe K (2011). On the existence of a possible A2A–D2-beta-Arrestin2 complex: A2A agonist modulation of D2 agonist-induced beta-arrestin2 recruitment. J Mol Biol.

[38] Borroto-Escuela DO, Romero-Fernandez W, Tarakanov AO, Gomez-Soler M, Corrales F, Marcellino D, Narvaez M, Frankowska M, Flajolet M, Heintz N, Agnati LF, Ciruela F, Fuxe K (2010). Characterization of the A2AR-D2R interface: focus on the role of the C-terminal tail and the transmembrane helices. Biochem Biophys Res Commun.

[39] Borroto-Escuela DO, Romero-Fernandez W, Wydra K, Zhou Z, Suder A, Filip M, Fuxe K (2020). OSU-6162, a Sigma1R ligand in low doses, can further increase the effects of cocaine self-administration on accumbal D2R heteroreceptor complexes. Neurotox Res.

[40] Ferre S, Karcz-Kubicha M, Hope BT, Popoli P, Burgueno J, Gutierrez MA, Casado V, Fuxe K, Goldberg SR, Lluis C, Franco R, Ciruela F (2002). Synergistic interaction between adenosine A2A and glutamate mGlu5 receptors: implications for striatal neuronal function. Proc Natl Acad Sci USA.

[41] Cabello N, Gandia J, Bertarelli DC, Watanabe M, Lluis C, Franco R, Ferre S, Lujan R, Ciruela F (2009). Metabotropic glutamate type 5, dopamine D2 and adenosine A2a receptors form higher-order oligomers in living cells. J Neurochem.

[42] Schwarzschild MA, Agnati L, Fuxe K, Chen JF, Morelli M (2006). Targeting adenosine A2A receptors in Parkinson's disease. Trends Neurosci.

[43] Carlsson A, Lindqvist M (1963). Effect of chlorpromazine or haloperidol on formation of 3methoxytyramine and normetanephrine in mouse brain. Acta Pharmacol Toxicol.

[44] Seeman P (1987). Dopamine receptors and the dopamine hypothesis of schizophrenia. Synapse.

[45] Lombroso PJ, Naegele JR, Sharma E, Lerner M (1993). A protein tyrosine phosphatase expressed within dopaminoceptive neurons of the basal ganglia and related structures. J Neurosci.

[46] Borroto-Escuela DO, Agnati LF, Bechter K, Jansson A, Tarakanov AO, Fuxe K (2015). The role of transmitter diffusion and flow versus extracellular vesicles in volume transmission in the brain neural-glial networks. Phil Trans R Soc London Ser B Biol Sci.

[47] Lombroso PJ, Ogren M, Kurup P, Nairn AC (2016). Molecular underpinnings of neurodegenerative disorders: striatal-enriched protein tyrosine phosphatase signaling and synaptic plasticity. F1000Research.

[48] Lambert LJ, Grotegut S, Celeridad M, Gosalia P, Backer LJ, Bobkov AA, Salaniwal S, Chung TD, Zeng FY, Pass I, Lombroso PJ, Cosford ND, Tautz L (2021). Development of a robust high-throughput screening platform for inhibitors of the striatal-enriched tyrosine phosphatase (STEP). Int J Mol Sci.

[49] Svenningsson P, Lindskog M, Rognoni F, Fredholm BB, Greengard P, Fisone G (1998). Activation of adenosine A2A and dopamine D1 receptors stimulates cyclic AMP-dependent phosphorylation of DARPP-32 in distinct populations of striatal projection neurons. Neuroscience.

[50] Hemmings HC, Greengard P, Tung HY, Cohen P (1984). DARPP-32, a dopamine-regulated neuronal phosphoprotein, is a potent inhibitor of protein phosphatase-1. Nature.

[51] Yan Z, Hsieh-Wilson L, Feng J, Tomizawa K, Allen PB, Fienberg AA, Nairn AC, Greengard P (1999). Protein phosphatase 1 modulation of neostriatal AMPA channels: regulation by DARPP-32 and spinophilin. Nat Neurosci.

[52] Dell'anno MT, Pallottino S, Fisone G (2013). mGlu5R promotes glutamate AMPA receptor phosphorylation via activation of PKA/DARPP-32 signaling in striatopallidal medium spiny neurons. Neuropharmacology.

[53] Popoli P, Pezzola A, Torvinen M, Reggio R, Pintor A, Scarchilli L, Fuxe K, Ferre S (2001). The selective mGlu(5) receptor agonist CHPG inhibits quinpirole-induced turning in 6-hydroxydopamine-lesioned rats and modulates the binding characteristics of dopamine D(2) receptors in the rat striatum: interactions with adenosine A(2a) receptors. Neuropsychopharmacology.

[54] Surmeier DJ, Ding J, Day M, Wang Z, Shen W (2007). D1 and D2 dopamine-receptor modulation of striatal glutamatergic signaling in striatal medium spiny neurons. Trends Neurosci.

[55] Salim H, Ferre S, Dalal A, Peterfreund RA, Fuxe K, Vincent JD, Lledo PM (2000). Activation of adenosine A1 and A2A receptors modulates dopamine D2 receptor-induced responses in stably transfected human neuroblastoma cells. J Neurochem.

[56] Orlando LR, Dunah AW, Standaert DG, Young AB (2002). Tyrosine phosphorylation of the metabotropic glutamate receptor mGluR5 in striatal neurons. Neuropharmacology.

[57] Krania P, Dimou E, Bantouna M, Kouvaros S, Tsiamaki E, Papatheodoropoulos C, Sarantis K, Angelatou F (2018). Adenosine A2A receptors are required for glutamate mGluR5- and dopamine D1 receptor-evoked ERK1/2 phosphorylation in rat hippocampus: involvement of NMDA receptor. J Neurochem.

[58] Borroto-Escuela DO, Tarakanov AO, Brito I, Fuxe K (2018). Glutamate heteroreceptor complexes in the brain. Pharmacol Rep.

[59] Gimenez-Llort L, Schiffmann SN, Shmidt T, Canela L, Camon L, Wassholm M, Canals M, Terasmaa A, Fernandez-Teruel A, Tobena A, Popova E, Ferre S, Agnati L, Ciruela F, Martinez E, Scheel-Kruger J, Lluis C, Franco R, Fuxe K, Bader M (2007). Working memory deficits in transgenic rats overexpressing human adenosine A2A receptors in the brain. Neurobiol Learn Mem.

[60] Chiodi V, Ferrante A, Ferraro L, Potenza RL, Armida M, Beggiato S, Pezzola A, Bader M, Fuxe K, Popoli P, Domenici MR (2016). Striatal adenosine-cannabinoid receptor interactions in rats over-expressing adenosine A2A receptors. J Neurochem.

[61] Su TP, Hayashi T (2001). Cocaine affects the dynamics of cytoskeletal proteins via sigma(1) receptors. Trends Pharmacol Sci.

[62] Matsumoto RR, Liu Y, Lerner M, Howard EW, Brackett DJ (2003). Sigma receptors: potential medications development target for anti-cocaine agents. Eur J Pharmacol.

[63] Fang X, Stachowiak EK, Dunham-Ems SM, Klejbor I, Stachowiak MK (2005). Control of CREB-binding protein signaling by nuclear fibroblast growth factor receptor-1: a novel mechanism of gene regulation. J Biol Chem.

[64] Romieu P, Phan VL, Martin-Fardon R, Maurice T (2002). Involvement of the sigma(1) receptor in cocaine-induced conditioned place preference: possible dependence on dopamine uptake blockade. Neuropsychopharmacology.

